# Butyrate regulates E-cadherin transcription, isoform expression and intracellular position in colon cancer cells

**DOI:** 10.1054/bjoc.1999.0899

**Published:** 1999-12-08

**Authors:** M Barshishat, S Polak-Charcon, B Schwartz

**Affiliations:** Institute of Biochemistry, Food Science and Nutrition, Faculty of Agricultural, Food and Environmental Quality Sciences, The Hebrew University of Jerusalem, Rehovot, 76100, Israel; Institute of Pathology, Sheba Medical Center, Tel-Hashomer, Israel

**Keywords:** adhesion proteins, E-cadherin, metastasis, colon cancer, butyrate

## Abstract

Cell-to-cell adhesion, an important event in differentiation, is impaired during advanced stages of tumorigenesis. In this study, we examined the possible regulation of cell-adhesion proteins by the differentiation agent butyrate in LS174T and HM7 cells, two types of human colon cancer cells that differ in their ability to produce mucin and colonize the liver of experimental animals. The more aggressive, high-mucin-producing cell line (HM7), a clone selected from LS174T cells, showed a scattered and undifferentiated ultramorphological appearance and low basal alkaline phosphatase activity; the proteins β-catenin and E-cadherin, as detected by immunostaining, were expressed in the cells’ nuclei. All of these properties were significantly less pronounced in the less aggressive, low-mucin-producing LS174T cells. In both cell lines, butyrate treatment enhanced cell-to-cell interaction, alkaline phosphate activity, translocation of β-catenin and E-cadherin from the nuclei to the membrane junctions, and transcription and translation of the 120-kDa E-cadherin isoform, but not of its 100-kDa isoform. Analysis of possible mechanisms of E-cadherin up-regulation revealed that butyrate induces the release of nuclear proteins from the E-cadherin promoter sequence, reducing transcription repression. We suggest that butyrate activates E-cadherin transcription through translocation of nuclear transcription factors bearing specific repressor activity. We surmise that abrogation of nuclear 100-kDa E-cadherin and β-catenin expression following butyrate treatment is related to the control of E-cadherin gene transcription. © 2000 Cancer Research Campaign


*British Journal of Cancer* (2000) **82**(1), 195–203
© 2000 Cancer Research Campaign
Article no. bjoc.1999.0899

### Butyrate regulates E-cadherin transcription, isoform expression and intracellular position in colon cancer cells

**M Barshishat[1], S Polak-Charcon[2] and B Schwartz[1]**

[1]Institute of Biochemistry, Food Science and Nutrition, Faculty of Agricultural, Food and Environmental Quality Sciences, The Hebrew University of Jerusalem, Rehovot 76100, Israel; [2]Institute of Pathology, Sheba Medical Center, Tel-Hashomer, Israel

**Summary** Cell-to-cell adhesion, an important event in differentiation, is impaired during advanced stages of tumorigenesis. In this study, we examined the possible regulation of cell-adhesion proteins by the differentiation agent butyrate in LS174T and HM7 cells, two types of human colon cancer cells that differ in their ability to produce mucin and colonize the liver of experimental animals. The more aggressive, high-mucin-producing cell line (HM7), a clone selected from LS174T cells, showed a scattered and undifferentiated ultramorphological appearance and low basal alkaline phosphatase activity; the proteins β-catenin and E-cadherin, as detected by immunostaining, were expressed in the cells' nuclei. All of these properties were significantly less pronounced in the less aggressive, low-mucin-producing LS174T cells. In both cell lines, butyrate treatment enhanced cell-to-cell interaction, alkaline phosphate activity, translocation of β-catenin and E-cadherin from the nuclei to the membrane junctions, and transcription and translation of the 120-kDa E-cadherin isoform, but not of its 100-kDa isoform. Analysis of possible mechanisms of E-cadherin up-regulation revealed that butyrate induces the release of nuclear proteins from the E-cadherin promoter sequence, reducing transcription repression. We suggest that butyrate activates E-cadherin transcription through translocation of nuclear transcription factors bearing specific repressor activity. We surmise that abrogation of nuclear 100-kDa E-cadherin and β-catenin expression following butyrate treatment is related to the control of E-cadherin gene transcription. © 2000 Cancer Research Campaign

**Keywords:** adhesion proteins; E-cadherin; metastasis; colon cancer; butyrate

E-cadherin belongs to a family of transmembrane glycoproteins that bind neighbouring epithelial cells in a homophilic and $Ca^{2+}$-dependent way (Takeichi, 1988, 1991; Behrens et al, 1989). The COOH domain is located within the cell and interacts with catenins, actin and other cytoskeletal proteins. The $NH_2$-terminal domain, located in the extracellular domain, contains the $Ca^{2+}$-binding sites essential for cell adhesion and binding specificity. The intracellular segment of E-cadherin interacts with α-, β-, and γ-catenins, which are related to the cytoskeleton proteins vinculin and actin. In many tumours, mutation and/or gene deregulation have been detected in all components of the cadherin–catenin complex. Any of these disorders, either at the genomic or protein level, affects the stability of the cadherin–catenin complex, which is directly evidenced by invasion and malignant progression (Gubiner, 1996). In colon carcinomas, down-regulation of E-cadherin expression (Frixen et al, 1991), considered a tumour-suppressor gene (Takeichi, 1993), or alternatively, elevation of β-catenin expression in cells bearing a mutant APC molecule, is oncogenic, because target genes related to tumour progression seem to be activated by this event (Rubinfeld et al, 1997). The E-cadherin gene promoter has been sequenced (Behrens et al, 1991) and several specific regions have been identified that contribute to tissue-specific activity and transcription regulation. These include a GC-rich region that generates basic epithelial promoter activity, a palindromic sequence E-pal that potentiates the activity of the proximal E-cadherin promoter, and a CAAT-box (Behrens et al, 1991).

Butyrate is a short-chain fatty acid formed in the gastrointestinal tract of mammals as a result of anaerobic bacterial fermentation of undigested dietary components, and avidly absorbed by the colonic epithelium. Although butyrate provides the preferred oxidative fuel for the normal colonocytes (Velazquez et al, 1996), neoplastic cell lines have been found to undergo terminal differentiation and/or apoptosis when treated with millimolar concentrations of n-butyrate, similar to the physiological intracolonic luminal concentrations (McBain et al, 1997). Treatment of mammalian cells in vitro with millimolar concentrations of butyrate has pleiotropic effects on cellular physiology, including changes in the cell membrane, cytoskeleton, the cell cycle and gene transcription (Hassing et al, 1997; Schwartz et al, 1998). Butyrate regulates transcription expression of multiple genes and has several effects on nuclear proteins that could modify gene expression (Nakano et al, 1997; Archer et al, 1998). The significance of these varied molecular effects is not well understood and differs among the many in vitro cell-line studies (Hassing et al, 1997; McBain et al, 1997; Nakano et al, 1997; Archer et al, 1998; Cuisset et al, 1998). One hypothesis is that butyrate acts as a co-factor for regulatory transcription proteins in the nuclei that are directly involved in gene expression (Hassing et al, 1997).

In the present study, we characterized the transcriptional mechanisms by which E-cadherin expression is up-regulated by butyrate in parental non-metastatic and metastatic cell lines.

*Received 26 January 1999*
*Revised 4 June 1999*
*Accepted 8 June 1999*

*Correspondence to:* B Schwartz

We demonstrate that butyrate down-regulates the binding of nuclear proteins to the promoter sequence of the E-cadherin gene, resulting in up-regulation of the adhesion protein's expression. Furthermore, we report that β-catenin is translocated from the nuclear to the intercellular junctions following butyrate treatment.

### MATERIALS AND METHODS

### Cell lines and culture conditions

LS174T is a colon cancer cell line derived from a well-differentiated human colonic adenocarcinoma (Tom et al, 1976). An HM7 cell variant of LS174T was previously selected for its capacity to produce high amounts of mucin (Kuan et al, 1987), relative to parental LS174T cells and shown to be highly metastatic in in vivo (Bresalier et al, 1995) and in vitro systems (Schwartz et al, 1992). Cells were maintained under a humidified atmosphere, 5% carbon dioxide, in Dulbecco's minimal essential medium supplemented with 10% fetal calf serum, 100 units $ml^{-1}$ penicillin and 100 μg $ml^{-1}$ streptomycin sulphate, and grown as undifferentiated multilayers under controlled conditions. Cells were treated with 2 mM butyrate for 9 days, as previously described (Schwartz et al, 1995*a*), to induce terminal differentiation.

All media and supplements were purchased from Beit Haemek (Biological Industries, Israel); biochemicals were purchased from Sigma Chemical Co. (St Louis, MO, USA) unless otherwise specified.

### Transmission electron microscopy

Cells were fixed in 2.5% glutaraldehyde in 0.1 M cacodylate buffer, pH 7.4, for 1 h. Fixed cells were removed with a rubber policeman, post-fixed in 1% $OsO_4$, dehydrated in graded ethanol solutions, and embedded in Epon. Ultrathin sections were contrasted with uranyl acetate and lead citrate and examined in a Jeol Jem 1200 EX II transmission electron microscope (Schwartz et al, 1995*a*).

### Alkaline phosphatase

Alkaline phosphatase activity was determined in cytosolic preparations by a standard spectrophotometric method using paranitrophenyl-phosphate as the substrate (Schwartz et al, 1995*a*).

### Western blot analysis

Cells were lysed, electrophoresed on a 10% sodium dodecyl sulphate (SDS) polyacrylamide gel, transferred to a nylon-transfer membrane (Amersham, Buckinghamshire, UK), blocked in TBS (10 mM Tris-base, 100 mM sodium chloride) containing 5% dry non-fat milk, incubated with mouse anti-human E-cadherin monoclonal antibody (Transduction Laboratories, Lexington, KY, USA), or β-catenin polyclonal antibody and subsequently incubated with secondary antibodies coupled to horseradish peroxidase. Both proteins were visualized using an enhanced chemiluminescence kit (ECL; Amersham, Buckinghamshire, UK).

### Immunocytochemistry

Cells were grown on chamber slides. Fixation and abrogation of endogenous peroxidase activity were performed in a solution of 3% hydrogen peroxide and 95% methanol. The fixed cells were incubated in an antibody directed to the extracellular domain of E-cadherin (DECMA-1) or in an anti-E-cadherin antibody directed to the intracellular domain (Transduction Laboratories, Lexington, KY, USA). Detection was performed with Vectastain Elit kit (Vector Laboratories, Burlingame, CA, USA). Counterstaining was performed with haematoxylin (Schwartz et al, 1995*b*).

### E-cadherin reverse transcription-polymerase chain reaction cDNA was synthesized from 5 μg of total RNA using a reverse transcription polymerase chain reaction (RT-PCR) kit (Promega, Madison, WI, USA). The primers used for the exon-9 sequence are: 5′ CTCAGCTCTGCTAGCAGTCTTG 3′ (forward), and 5′ GATGAACACCTGTGAGGAG 3′ (reverse). The PCR amplification conditions were: 30 s, 94°C; 30 s, 55°C; 30 s, 72°C, for 30 cycles. PCR products were separated by agarose gel; molecular weight was confirmed with a 123-bp DNA ladder (Gibco-BRL, Paisley, UK).

### Human serum albumin RT-PCR

cDNA was synthesized from 5 μg of total RNA using an RT-PCR kit (Promega, Madison, WI, USA). The primers used for the PCR amplification were 5′CCAAGAAAGTACCCCAAGTGTC 3′ (forward), and 5′ TGTTTCATCGACTTCCAGAGC 3′ (reverse). PCR amplification conditions were: one cycle at 94°C for 2.5 min, ten cycles at 40 s, 94°C; 30 s, 55°C; 20 cycles at 30 s, 94°C; 20 s, 53°C, 1 min, 72°C and then one cycle at 72°C for 5 min. PCR products were separated by agarose gel; molecular weight was confirmed with a 123-bp DNA ladder (Gibco-BRL, Paisley, UK).

### Amplification of E-cadherin promoter by PCR

A 330-bp fragment from the human E-cadherin promoter was amplified (from 721 to 1080, GenBank database L34545) (Bussemakers et al, 1994) using primers: upstream 5′-ACTCAGCCAAGTGTAAAAG-3′, downstream 5′-TCACAGGTGCTTTGCAGTT-3′. The PCR programme was: 35 cycles of 94°C; 45 s, 46°C; 45 s, 72°C and one cycle at 72°C for 5 min. The PCR products were separated on an agarose gel, their molecular weight confirmed with a 123-bp DNA ladder, and completely sequenced to ensure no unexpected mutations.

### Gel shift mobility assay

Nuclear proteins were extracted from $5 \times 10^6$–$10^7$ control or butyrate-treated cells as previously described (Andrews et al, 1991). Nuclear proteins (10 μg) were preincubated with poly dI-dC and then in reaction buffer (100 mM Tris–HCl pH 7.9, 25 mM magnesium chloride ($MgCl_2$), 2 mM EDTA, 40% glycerol, 200mM potassium chloride, 0.2% NP-40 and 2 mM dithiothreitol, and the 330-bp amplified DNA fragment of E-cadherin promoter sequence was end-labelled with [γ-$^{32}$P]ATP and T4 polynucleotide kinase (BioLab New England, Beverly, MA, USA). The samples were separated by electrophoresis in a 2.4% acrylamide gel. The gel was dried and subjected to autoradiography (Carey, 1991).

### β-catenin detection by immunofluorescence staining

Cells were grown on coverslips for 9 days with or without butyrate. The coverslips were rinsed in a phosphate-buffered

© 2000 Cancer Research Campaign

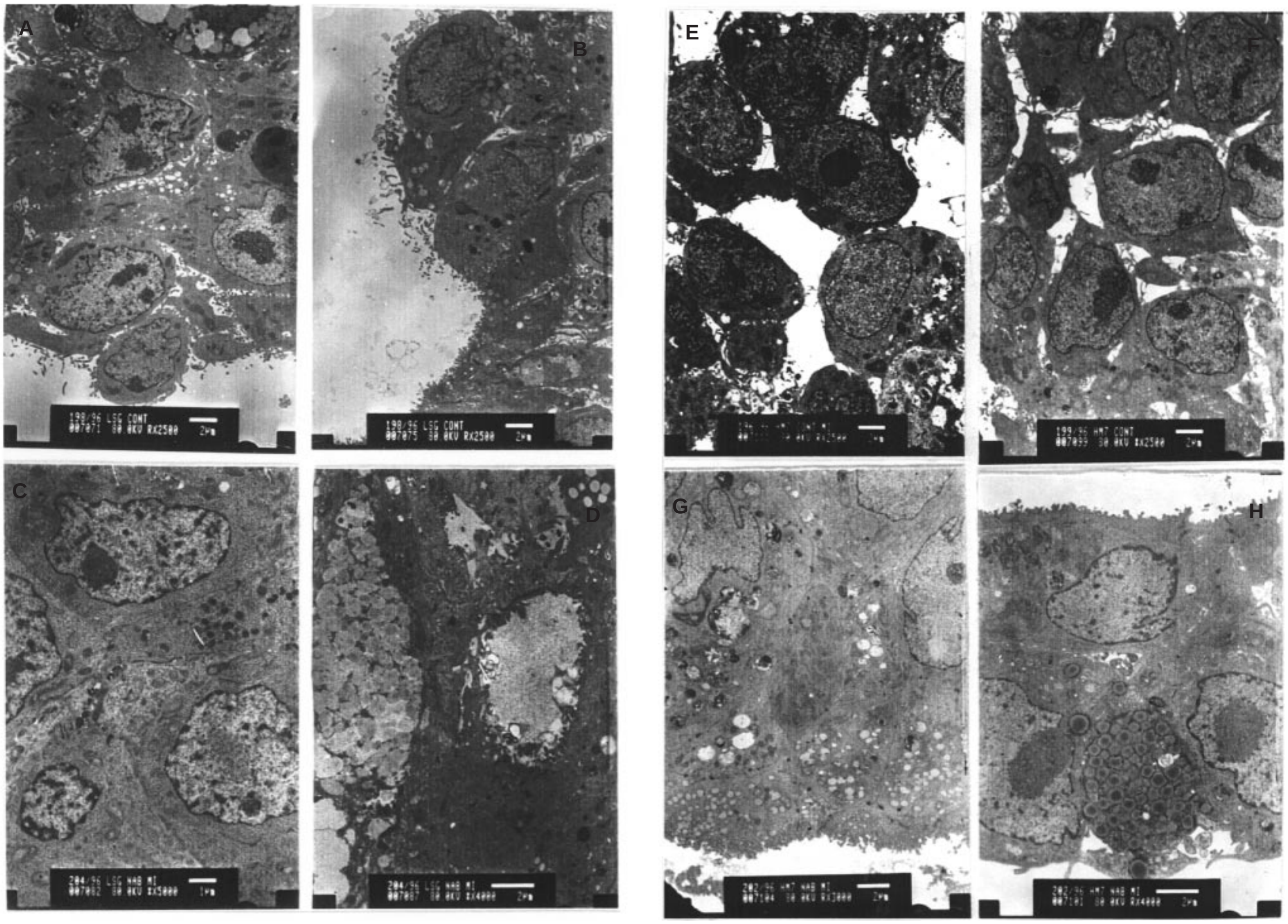

**Figure 1** (**A–D**) Representative ultrastructural analysis of LS174T cells. (**A, B**) Partially differentiated untreated cells, showing undeveloped brush-border membranes and few contact points between the cells. (**C, D**) Butyrate-treated LS174T cells, polarized and adhering to each other. Typical signs of differentiation are observed, e.g. appearance of goblet cells (**D**). (**E–H**) Representative ultrastructural analysis of HM7 cells. (**E, F**) Untreated cells show no intercellular contacts, and a high nuclei/cytoplasm ratio. Butyrate-treated HM7 cultures (**G, H**) show well-organized epithelial cells, highly polarized and notable intercellular contacts are evident, resulting in a tissue-like appearance of this culture saline (PBS) solution (containing 1 mM $MgCl_2$ and 0.1 mM calcium chloride). Fixation was performed with cooled methanol (–20°C) for 10 min and 1 min in cooled acetone (–20°C). The fixed cells were incubated with a human polyclonal anti-β-catenin antibody. The secondary antibody used was rhodamine-labelled goat anti-rabbit IgG (Promega, Madison, WI, USA). Counterstaining was performed with 4′6-diamidino-2-phenylindole hydrochloride (DAPI) diluted in anti-fade solution (0.7 μg $ml^{-1}$).

### RESULTS

### Characteristics of cell differentiation

We studied the effect of butyrate treatment by means of differentiation-related criteria: ultramorphological analysis and alkaline phosphatase activity.

Ultrastructurally, LS174T control cells showed scattered signs of differentiation, sparse brush-border membranes, and minimal cell-to-cell contacts (Figure 1 A,B). In contrast, butyrate-treated cells showed well-defined cell-to-cell contacts, well-developed junctional complexes (Figure 1 C,D) and cells bearing a goblet-like morphology. The highly metastatic HM7 cells grown under

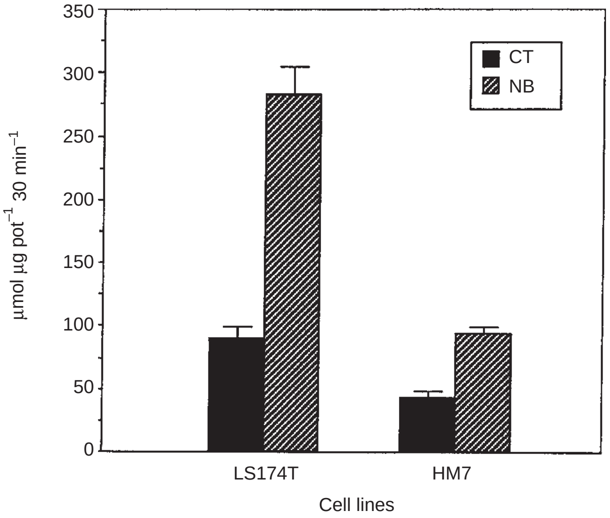

**Figure 2** Alkaline phosphatase activity in LS174T and HM7 cells cultured for 9 days in control media (CT) (■) is low. Cells incubated in media containing 2 mM butyrate (NB) (▨) show significant ($P < 0.001$) enhancement of alkaline phosphatase activity relative to their respective control cultures. The values represent the mean ± s.e.m. of six individual determinations

© 2000 Cancer Research Campaign

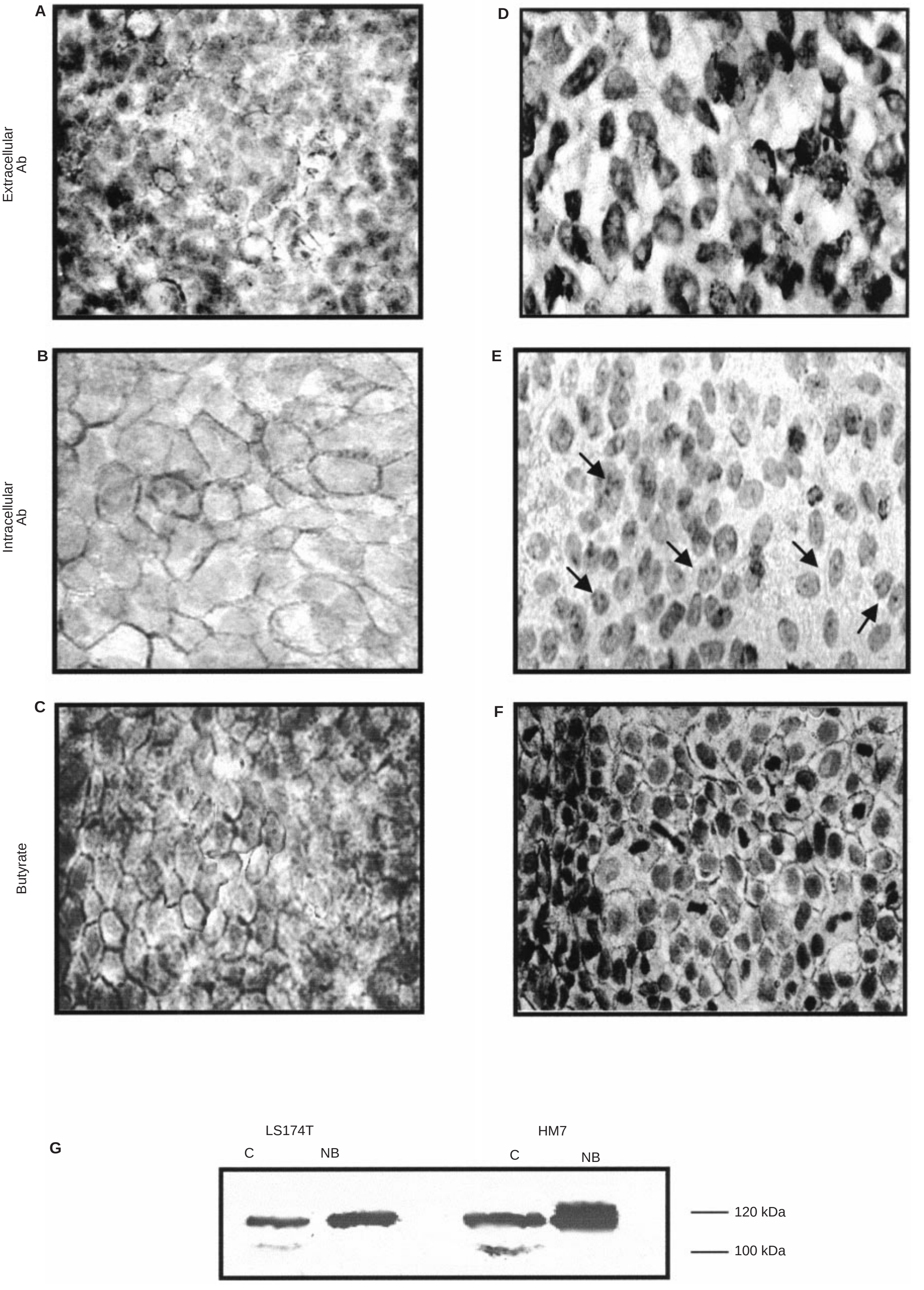

**Figure 3** (**A–F**) Immunohistochemical E-cadherin analysis of LS174T and HM7 cells. Cells were stained with extracellular and intracellular E-cadherin-directed antibodies. (**A, B, C**) LS174T cells stained with both antibodies. Control cells stain positively for E-cadherin and butyrate accentuates this pattern. (**D**) Extracellular staining is not detected in HM7 cells. (**E**) Intracellular staining of HM7 cells shows expression in the membrane and/or also in the nuclei (see arrows). (**F**) Butyrate-treated HM7 cells: E-cadherin is expressed strongly in the extracellular domain. (**G**) Western blot analysis of E-cadherin protein. Control (CT) LS174T cells and HM7 cells exhibit the 100- and the 120-kDa isoforms. Butyrate treatment (NB) causes the disappearance of the 100-kDa E-cadherin species in both cell lines. The effect is more pronounced in HM7 cells. The results represent one representative experiment of four identical performed

*© 2000 Cancer Research Campaign*

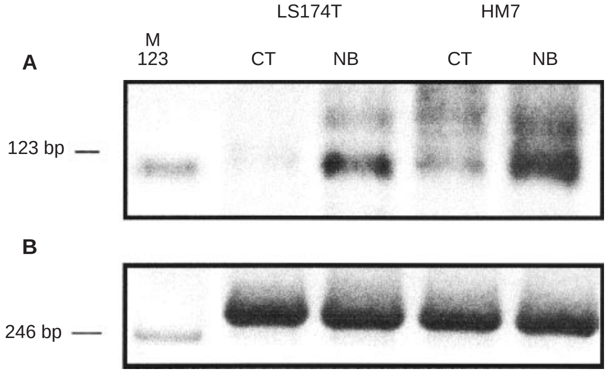

**Figure 4** RT-PCR assay to analyse the expression of: (**A**) E-cadherin cDNA 123-bp products. RNA was harvested from LS174T and HM7 cells, untreated (CT) or treated with butyrate (NB). Butyrate induced significant up-regulation of E-cadherin expression in both cell lines. M: 123-bp marker. (**B**) The housekeeping gene human serum albumin, to ensure equal RNA loading. The figure is one representative experiment out of four performed

E–cadherin promoter sequence

**Figure 5** (**A**) A gel mobility assay was carried out with nuclear extracts from butyrate-treated (NB) or untreated (CT) colon cancer cells. A 123-bp sequence of exon-9 was used as a control specific-binding experiment. Similar results were obtained in eight additional experiments. (**B**) The 330-bp E-cadherin promoter sequence containing the E-pal palindromic element (bold), the CAAT-box (italic) and Sp1 consensus sequence (underlined) was $\gamma$-$^{32}$P end-labelled and used for the gel mobility assay control conditions were sparse and intracellular junctions were absent. These cells had a high nucleus/cytoplasm ratio. The cultures were not polarized and no contact between the cells was observed (Figure 1 E,F). Following butyrate treatment, striking morphological changes emerged: the cells showed polarized morphology and well-defined cell-to-cell contacts, junctional complexes and brush-border membranes (Figure 1 G,H).

Alkaline phosphatase activity was determined in control LS174T and HM7 colon cancer cells. Basal activity was higher in the LS174T cells than in HM7 (Figure 2). Following 9 days of butyrate treatment, a threefold rise in enzymatic activity was observed in the LS174T cells relative to their untreated counterparts. Although the HM7 cells showed a significant, twofold increase in enzymatic activity as compared to controls, absolute values were lower than those of the treated LS174T cells (Figure 2).

### E-cadherin immunocytology

Immunocytochemical staining of the extracellular domain of E-cadherin in LS174T control cells revealed both diffuse and typical extracellular organization of protein expression (Figure 3A). Reaction with antibody directed to the intracellular domain of E-cadherin, the same used for the Western analysis, showed mostly membrane staining in LS174T control cells, similar to the extracellular antibody, and minimal nuclear staining (Figure 3B). Complete translocation of this diffuse and faint nuclear staining to the extracellular junctions was observed in LS174T cells after butyrate treatment, as evidenced by immunocytochemical reactions with both antibodies (Figure 3C).

E-cadherin expression in control HM7 cells was sporadic, with a predominant non-specific staining pattern using antibody to the extracellular domain, and was evident in small groups of cells (Figure 3D). A high amount of nuclear staining was detected in HM7 control cells when antibody directed to the intracellular domain was used (Figure 3E). In comparison, striking difference was observed in HM7 cells treated with butyrate. Butyrate-treated cells showed typical extracellular staining of E-cadherin, mimicking a delicate net. Similar immunocytochemical results were obtained with both antibodies. Nuclear staining was not detected in HM7 cells after butyrate treatment (Figure 3F). Similar staining patterns were obtained with both the intracellular (data not shown) and extracellular staining (Figure 3 C,F).

### Regulation of E-cadherin expression by butyrate

Figure 3G shows a Western blot analysis of E-cadherin protein from LS174T and HM7 cells untreated or treated with butyrate, using antibody directed to the intracellular domain of the protein. Untreated LS174T and HM7 cells showed full-size (120 kDa) and a smaller size (100 kDa) E-cadherin reactive protein. The expression of the low-molecular-weight isoform was higher in the aggressive HM7 cells than in the LS174T parental cells. Butyrate treatment up-regulated the expression of the 120-kDa isoform and completely abolished that of the 100-kDa isoform. This effect was more pronounced in HM7 cells than in the parental LS174T cells.

### RNA expression detected by RT-PCR

The expression of E-cadherin was analysed by RT-PCR (Figure 4A). Although the methodology is semi-quantitative, the level of

*© 2000 Cancer Research Campaign*

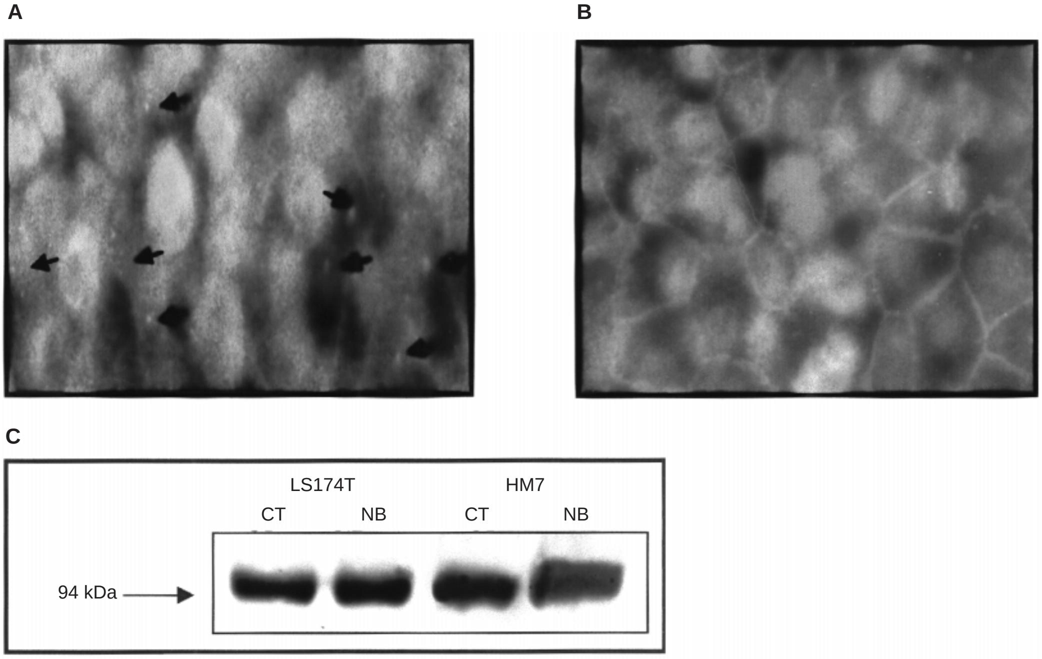

**Figure 6** Representative immunofluorescent staining (**A, B**) and Western blot analysis (**C**) for the detection of β-catenin using a polyclonal anti-β-catenin antibody. (**A**) Control HM7 cells show sporadic and non-specific intracellular staining; significant nuclear expression is evident (see arrows). (**B**) HM7 cells treated with butyrate: β-catenin staining is evident exclusively at the intercellular borders. No immunofluorescent staining is detectable within the nuclei. Similar findings were obtained for control and butyrate-treated LS174T cells (data not shown). (**C**) β-catenin expression was not affected by butyrate treatment as detected by Western blot analysis. The same was found for LS174T cells expression could be clearly established because transcription of the E-cadherin gene was significantly up-regulated in the presence of butyrate in HM7 cell cultures. This was also the case for butyrate-treated LS174T cells. To ensure that closely matched amount of cDNA from RNA were used in the reaction a portion of the housekeeping gene HSA was amplified in parallel with the E-cadherin primers. Equal amounts of HSA were detected in both cell lines with ore without butyrate treatment (Fig 4B).

### Gel mobility assay

The ability of nuclear proteins to bind to a 330-bp segment of human E-cadherin promoter and to the 123-bp sequence of exon 9 was assessed (Figure 5). Nuclear proteins did not bind to exon 9. The highest binding activity of nuclear proteins to the E-cadherin promoter was detected when the extract was derived from HM7 control cells. This binding capacity was abrogated by butyrate treatment. LS174T cells showed a similar pattern of binding activity following butyrate treatment; however, its overall intensity was lower as compared to HM7 cells.

### β-catenin translocation after butyrate treatment

β-catenin was detected in both untreated LS174T cells (not shown) and HM7 cells (Figure 6). In LS174T cells, butyrate treatment did not affect β-catenin immunostaining. In control HM7 cells, β-catenin was sporadic; a non-specific intracellular staining pattern was predominant and several β-catenin immunoresponsive spots were detected within the nuclei (Figure 6A, see arrows). However, when HM7 cells were treated with butyrate, dramatic translocation of β-catenin out of the nucleus took place and strong typical intercellular junctional staining was evident (Figure 6B). Western blot analysis revealed that the level of β-catenin expression is not affected by butyrate treatment in either LS174T or HM7 cells (Figure 6C).

### DISCUSSION

We studied two colon cancer cell lines differing in a number of phenotypes: (i) a parental colon cancer cell line (LS174T) characterized by its low capacity to produce mucin concomitant with its low ability to colonize the liver in experimental animal models, and (ii) a high-mucin selected clone (HM7) characterized by high in vivo and in vitro invasive characteristics (Schwartz et al, 1992; Bresalier et al, 1995). Ultramorphological analysis of both cultures reveal that LS174T cells show scattered signs of differentiation and intercellular contacts and HM7 cells without any sign of intercellular contact.

Alkaline phosphatase activity is considered to be a valid marker of colonic differentiation (Schory et al, 1994; Schwartz et al, 1995*a*). Consistent with this view, the lowest alkaline phosphatase activity was measured in the untreated, undifferentiated HM7 cells. Higher levels were observed in partially differentiated LS174T cells. We then compared the responses of the two colon cancer cell lines to butyrate treatment. In both cultures, intercellular contacts were enhanced after the treatment. Ultrastructurally, the invasive clone responded more strongly to butyrate treatment. The intercellular adhesive capacity, particularly in HM7 cells, changed dramatically. Butyrate-treated HM7 cells showed a polarized morphology, brush-border structures typical to differentiated colonocytes, differentiated goblet cells and, most significantly, the appearance of many tight connections between the cells.

Takeichi (1993) reported that decreased expression of E-cadherin is correlated with de-differentiation and metastasis in

 © 2000 Cancer Research Campaign

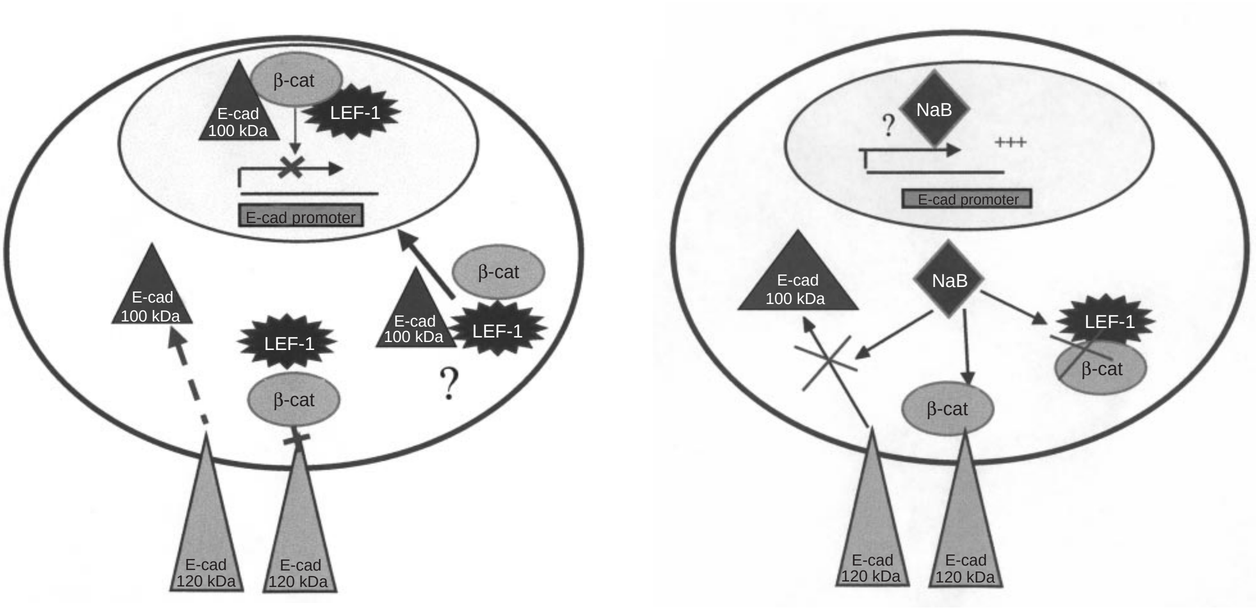

**Figure 7** Hypothetical mechanism of action of butyrate on HM7 colon cancer cells. (**A**) Control conditions, (**B**) effect of butyrate several poorly differentiated carcinomas. Mutation or transcriptional down-regulation are major mechanisms leading to decreased E-cadherin expression (Becker et al, 1994; Henning et al, 1995). The cell-adhesion molecule E-cadherin is a 120-kDa cell-surface glycoprotein, from which an intracellular cytosolic 100-kDa soluble fragment can be released (Gofuku et al, 1998). The parental LS174T cells and the highly invasive HM7 subclone expressed both the full-size and the intracellular cytosolic tryptic 100-kDa soluble protein isoform. Treatment of both cell types with butyrate induced the disappearance of the 100-kDa isoform and up-regulation of the 120-kDa full-size protein. The extent of the up-regulation response differed between the parental LS174T cells and the highly invasive HM7 subclone with butyrate treatment inducing a higher up-regulation of E-cadherin in HM7 cells. It can be surmised that the parental, less aggressive LS174T cell lines are in a more advanced stage of differentiation, which is merely accentuated by the treatment.

We performed an immunohistochemical analysis with two different antibodies to E-cadherin, directed to the intracellular or the extracellular domain. Extracellular staining showed a significant amount of the E-cadherin expression in untreated LS174T cells to be confined to the intercellular junctions; butyrate treatment only emphasized this pattern. The expression of E-cadherin was sporadic, faint and diffuse in control HM7 cells. In contrast, butyrate-treated HM7 cells showed the typical extracellular staining of E-cadherin, resembling a delicate net, and increased levels of E-cadherin expression accompanied by a clear intercellular redistribution of the protein. The intracellular staining showed E-cadherin expression in untreated LS174T cells to be confined to the intercellular junctions, with little localization in the nuclei, whereas in HM7 cells, E-cadherin was sporadic, faint, diffuse and mostly localized in the nuclei. These findings suggest that the increased contact between butyrate-treated LS174T and HM7 cells is associated with the enhanced E-cadherin expression.

To understand the mechanism by which butyrate up-regulates E-cadherin expression, we first investigated the possibility that treatment of the cells with the short fatty acid causes selection for a cell subpopulation presenting the normal E-cadherin gene. We assumed that the non-treated cultures contained some cells expressing the normal adhesion protein gene and some bearing the putative mutation lacking a G nucleotide at the exon/intron 9 border of the E-cadherin gene known to encode the calcium-binding site (Becker et al, 1994). Analysis of this possible specific and common mutation by nested PCR methodology revealed that butyrate does not select a mutant-free cell culture (data not shown).

Another approach was to search for events, which do not involve irreversible genetic alterations. Increased transcriptional activity is a major mechanism leading to increased protein expression (Bussemakers et al, 1994). We have shown that butyrate treatment of LS174T parental cell lines and HM7 cells specifically induces E-cadherin mRNA and protein. The human E-cadherin gene promoter comprises regulatory regions composed of various consensus sequences known as cis-acting elements, such as E-pal, the CAAT-box and a GC-rich region (Figure 6B), that contribute positively or negatively to promoter activity (Walsh and Doherty, 1993). We amplified a 330-bp segment of the promoter, including all the aforementioned consensus sequences and identified cell-specific band-retardation shifts within this sequence. The binding of the nuclear proteins was most notable in control HM7 cells, less pronounced in control LS174T cells, and significantly reduced in butyrate-treated LS174T and HM7 cells. Sequential control experiments not including cis-acting elements (exon-9) did not exhibit similar binding of nuclear proteins, indicating specificity of the promoter region analysed. These findings suggest that the enhanced binding of nuclear proteins to the 330-bp promoter region in LS174T and HM7 cells is associated with transcriptional repression of the E-cadherin gene. Butyrate may induce displacement of those nuclear proteins that bear repressor activity. Henning et al (1995) demonstrated that loss of differentiation of a wide range of carcinoma cells, as revealed by down-regulation of E-cadherin expression, directly correlates with specific changes in the chromatin structure. They observed that in undifferentiated cells, chromatin acquires a more condensed conformation,

© 2000 Cancer Research Campaign 

preventing the free approach of transcription factors to essential elements within the promoter. Upon butyrate treatment, global modification of chromatin proteins, such as hyperacetylation of histones, may result in significant interactions with these regulatory regions, enabling constitutively expressed, general transcription factors to gain access to their recognition sequences, recruit other factors, and activate transcription by direct activation or depression. In this study, we show that E-cadherin gene expression is up-regulated by butyrate treatment and that the short-chain fatty acid interacts with nuclear proteins which ultimately allow gene transcription and enhanced protein expression.

We show that β-catenin is differentially compartmentalized in the different cell lines, both in controls and after butyrate treatment. β-catenin, localized to the nuclei of control cells, was more evident in the high-mucin (HM7) cell line than in the parental one (LS174T). Butyrate induced dramatic translocation of β-catenin from the nuclei into the intercellular junctional domain in both cell lines. Treatment with the short-chain fatty acid did not, however, affect intracellular β-catenin protein concentration. The presence of nuclear β-catenin may indicate that the protein, by itself or in association with lymphoid-enhancing binding factors family (LEF/TCF), can transcriptionally regulate gene activity (Simcha et al, 1998*a*). Alternatively, transmembrane localization of β-catenin, due to strong binding to E-cadherin rather than to LEF, may result in inhibited transactivation activity and the oncogenic nature of the adhesion protein (Simcha et al, 1998*a*).

The mechanism by which butyrate induces the specific translocation of β-catenin from the nucleus to the transmembranal domain has yet to be elucidated; however, it may be related to the presence of the 100-kDa cytoplasmatic domain of E-cadherin in the untreated cell lines. Simcha et al (1998*b*) showed that transfection with the cytoplasmic tail of E-cadherin inhibits transactivation by competing with LEF-1 in the nucleus for β-catenin binding. Their results indicate that the cytoplasmic partners of β-catenin, E-cadherin and α-catenin, can sequester β-catenin and inhibit its transcriptional activity, thereby antagonizing its oncogenic action.

We assume that this cytoplasm E-cadherin isoform is able to bind β-catenin as do the LEF/TCF nuclear transcription factors. Once this complex is obtained, the 100-kDa protein may act as a nuclear protein allowing the translocation of β-catenin to the nuclei and subsequently regulate the expression of cancer-related genes. Butyrate treatment induced the total abrogation of 100-kDa E-cadherin expression concomitant with significant up-regulation of the 120-kDa full-size glycoprotein's expression. Therefore, in treated cells, β-catenin does not have its 'nuclear translocating partner', i.e. the 100-kDa E-cadherin isoform. On the contrary, there is enhanced expression of the transmembranal protein that sequesters β-catenin to the membranous domain (see a scheme of our hypothesis in Figure 7A and B). In the cell lines studied, the varying extent of β-catenin nuclear translocation could result from quantitative differences in 100-kDa E-cadherin expression. Both cell lines differ in mucin content and express MUC1 (unpublished results). Li et al (1998) demonstrated that the cytoplasmic domain of MUC1 interacts with β-catenin. The higher the expression of the MUC1 glycoprotein, the lower the interaction of β-catenin with the E-cadherin cell-adhesion molecule, allowing free β-catenin to reach the nuclei. It would appear that in high-mucin-producing cell lines, less β-catenin molecules interact with the 120-kDa transmembranal E-cadherin and are thus free to be translocated into the nucleus. In the low-mucin-producing cells, lower numbers of β-catenin molecules reach the nucleus.

Cumulatively, our findings suggest that treatment with specific substances, such as butyrate, can convert a passive essential promoter of a tumour-suppressor gene, such as the E-cadherin promoter, to an active form, thereby affecting the entire metastatic process. Studies showing these direct effects are in progress in our laboratory.

### REFERENCES

Andrews NC and Faller DV (1991) A rapid micropreparation technique for extraction of DNA-binding proteins from limiting numbers of mammalian cells. *Nucleic Acids Res* **19**: 2499

Archer SY, Meng S, Shai A and Hodin R (1998) p21$^{WAF1}$ is required for butyrate-mediated growth inhibition of human colon cancer cells. *Proc Natl Acad Sci USA* **95**: 6791–6796

Becker KF, Atkinson MJ, Reich U, Becker I, Nekarda H, Siewert JR and Hofler H (1994) E-cadherin gene mutations provide clues to diffuse type gastric carcinomas. *Cancer Res* **54**: 3845–3852

Behrens J, Mareel MM, Van Roy FM and Birchmier W (1989) Dissecting tumor cell invasion: epithelial cells acquire invasive properties after loss of uvomorulin-mediated cell–cell adhesion. *J Cell Biol* **108**: 2435–2447

Behrens J, Lowrick O, Klein-Hitpass L and Birchmeier W (1991) The E-cadherin promoter: functional analysis of G~C-rich region and an epithelial cell-specific palindromic regulatory element. *Proc Natl Acad Sci USA* **88**: 11495–11499

Bresalier RS, Schwartz B, Kim YS, Du Q-H, Kleinman HK and Sullam PM (1995) The laminin α1 chain Ile-Lys-Val-Ala-Val (IKVAV)-containing peptide promotes liver colonization by human colon cancer cells. *Cancer Res* **55**: 2476–2480

Bussemakers MJG, Giroldi LA, Van Bokhoven A and Schalken JA (1994) Transcriptional regulation of the human E-cadherin gene in human prostate cancer cell lines: characterization of the human E-cadherin gene promoter. *Biochem Biophys Res Commun* **203**: 1284–1290

Carey J (1991) Protein-DNA-interaction. Gel retardation. In: *Methods in Enzymology*, Sauer RT (ed), pp. 103–117. Academic Press: San Diego

Cuisset L, Tichonicky L and Delpech M (1998) A protein phosphatase is involved in the inhibition of histone deacetylation by butyrate. *Biochem Biophys Res Commun* **246**: 760–764

Gofuku J, Shiozaki H, Doki Y, Inoue M, Hirao M, Fukuchi N and Monden M (1998) Characterization of soluble E-cadherin as a disease marker in gastric cancer patients. *Br J Cancer* **78**: 1095–1101

Gubiner BM (1996) Cell adhesion: the molecular basis of tissue architecture and morphogenesis. *Cell* **84**: 345–357

Hassing CA, Tong JK and Schriber SL (1997). Fiber-derived butyrate and the prevention of colon cancer. *Crosstalk Chemistry Biology* **4**: 783–789

Hennig G, Behrens J, Truss M, Frisch S, Reichmann E and Birchmeier W (1995) Progression of carcinoma cells is associated with alteration in chromatin structure and factor binding at the E-cadherin promoter in vivo. *Oncogene* **11**: 475–484

Kuan SF, Byrd JC, Basbaum CB and Kim YS (1987) Characterization of quantitative mucin variants from a colon cell line. *Cancer Res* **47**: 5715–5724

Li Y, Bharti A, Chen D, Gong J and Kufe D (1998) Interaction of glycogen synthase kinase 3b with the DF3/MUC1 carcinoma-associated antigen and β-catenin. *Mol Cell Biol* **18**: 7216–7224

McBain JA, Eastman A, Nobel S and Mueller GC (1997) Apoptotic death in adenocarcinoma cell lines induced by butyrate and other histone deacetylase inhibitors. *Biochem Pharmacol* **53**: 1357–1368

Nakano K, Mizuno T, Sowa Y, Orita T, Yoshino T, Okuyama Y, Fujita T, Ohanti-Fujita N, Matsukawa Y, Tokino T, Yamagishi H, Oka T, Nomura H and Sakai Y (1997) Butyrate activates WAF1/Cip 1 gene promoter through SP1 sites in a p53-negative human colon cancer cell line. *J Biol Chem* **272**: 22199–22206

Robyr D and Wolffe P (1998) Hormone action and chromatin remodeling. *Cell Mol Life Sci* **54**: 113–124

Rubinfeld B, Albert I, Porfiri E, Munemitsu S and Polakis P (1997) Loss of β-catenin regulation by the APC tumor suppressor protein correlates with loss of structure due to common somatic mutations of the gene. *Cancer Res* **57**: 4624–4630

Schory PC, Rustig KA, Ikonomu E, Liu XP, Polito J, Andry C and O'Keane JC (1994) Growth and intestinal differentiation are independently regulated in HT29 colon cancer cells. *J Cell Phys* **161**: 111–123

Schwartz B, Bresalier RS and Kim YS (1992) Role of mucin in cancer metastasis. *Int J Cancer* **52**: 60–65

 © 2000 Cancer Research Campaign

Schwartz B, Lamprecht SA, Polak-Charcon S, Niv Y and Kim YS (1995*a*) Induction of the differentiated phenotype in human colon cancer cells is associated with the attenuation of subcellular tyrosine phosphorylation. *Oncol Res* **7**: 277–287

Schwartz B, Benharroch D, Prinsloo I, Cagnano E and Lamprecht SL (1995*b*) Phosphotyrosine, p62 c-myc and p21 c-Ha-ras proteins in colonic epithelium of normal and dimethylhydrazine-treated rats: an immunohistochemical analysis. *Anticancer Res* **15**: 211–218

Schwartz B, Avivi-Green C and Polak-Charcon S (1998) Sodium butyrate induces retinoblastoma protein dephosphorylation, p16 expression and growth arrest of colon cancer cells. *Mol Cell Biochem* **188**: 21–30

Simcha I, Shtutman M, Salomon D, Zorinsky J, Sadot E, Geiger B and Ben-Zeev A (1998*a*) Differential nuclear translocation and transactivation potential of β-catenin and plakoglobin. *J Cell Biol* **141**(6): 1433–1448

Simcha I, Shtutman M, Salomon D, Zorinsky J, Sadot E, Geiger B and Ben-Zeev A (1998*b*) Differential nuclear translocation and transactivation potential of β-catenin and plakoglobin. *5th IUBMB Conference on The Biochemistry of Health and Diseases*, Jerusalem, Israel, 18–22 October (Abstract)

Takeichi M (1988) The cadherins: cell–cell adhesion molecules controlling animal morphogenesis. *Development* **102**: 639–655

Takeichi M (1991) Cadherin cell adhesion receptors as morphogenetic regulator. *Science* **251**: 1451–1455

Takeichi M (1993) Cadherins in cancer: implications for invasion and metastasis. *Curr Opin Cell Biol* **5**: 806–811

Tom BH, Rutzky LP, Jakstys MM, Oyasu R, Kaye CI and Kahan BD (1976) Human colonic adenocarcinoma cells. I. Establishment and description of a new line. *In Vitro* **12**: 180–191

Walsh FS and Doherty P (1993) Factors regulating the expression and function of calcium-independent cell adhesion molecules. *Curr Opin Cell Biol* **5**: 791–796

Velazquez OC, Lederer HM and Rombeau JL (1996) Butyrate and the colonocyte: implications for neoplasia. *Dig Dis Sci* **41**: 727–739

*© 2000 Cancer Research Campaign*
